# Exploring the potential of vagus nerve stimulation in treating brain diseases: a review of immunologic benefits and neuroprotective efficacy

**DOI:** 10.1186/s40001-023-01439-2

**Published:** 2023-10-19

**Authors:** Zeping Jin, Jing Dong, Yang Wang, Yunpeng Liu

**Affiliations:** 1 Department of Neurosurgery, Beijing Chao-Yang Hospital, Capital Medical University, Beijing, People’s Republic of China; 2 Department of Medical Engineering, Tsinghua University Yuquan Hospital, Beijing, People’s Republic of China

**Keywords:** Vagus nerve stimulation, Neuroinflammation, Brain diseases, Neural protection

## Abstract

The vagus nerve serves as a critical connection between the central nervous system and internal organs. Originally known for its effectiveness in treating refractory epilepsy, vagus nerve stimulation (VNS) has shown potential for managing other brain diseases, including ischaemic stroke, traumatic brain injury, Parkinson's disease, and Alzheimer's disease. However, the precise mechanisms of VNS and its benefits for brain diseases are not yet fully understood. Recent studies have found that VNS can inhibit inflammation, promote neuroprotection, help maintain the integrity of the blood-brain barrier, have multisystemic modulatory effects, and even transmit signals from the gut flora to the brain. In this article, we will review several essential studies that summarize the current theories of VNS and its immunomodulatory effects, as well as the therapeutic value of VNS for brain disorders. By doing so, we aim to provide a better understanding of how the neuroimmune network operates and inspire future research in this field.

## Introduction

Most diseases of the central nervous system (CNS) are accompanied by functional impairment, sometimes leading to life-long neural disability [1, 2]. To date, the major strategies employed against the progression of brain diseases include pharmaceutical and surgical management, mainly targeting the lesion itself, cognitive behavioural rehabilitation. However, these methods, in most cases, cannot completely ameliorate functional damage after the onset of CNS diseases [3, 4]. Therefore, novel strategies are currently being studied to further improve the overall prognosis of CNS diseases.

The vagus nerve is the major neural component connecting the CNS and internal organs, including the gastrointestinal tract, and is composed of approximately 80% afferent and 20% efferent fibres [5]. Vagus nerve stimulation (VNS), a neuromodulatory therapy, is widely known as the first-line treatment for drug-resistant epilepsy [6]. In recent years, VNS has been found to have potential in other neural disorders. For example, in 2005, VNS was approved as an adjuvant therapy for major depression [7]. Recently, in patients with poststroke disability of the arms, motor function was significantly improved after rehabilitation therapy accompanied by VNS [8]. In addition, some studies found that patients with Alzheimer’s disease achieved improved cognitive performance according to their Alzheimer's Disease Assessment Scale-cognitive subscale (ADAS-cog) and Mini-Mental State Examination (MMSE) scores after VNS treatment for one year [9]. With the deepening of research, the application of VNS has been extended to the treatment of more CNS diseases [10–14].

Neuroinflammation is known as a major issue for the poor outcome of brain diseases, and attention has been drawn to the anti-inflammatory effects of VNS. In the current article, we intend to explore the different pathways and mechanisms of VNS by reviewing relevant studies and to emphasize the homeostatic effects of VNS on brain diseases.

A literature review was conducted until February 2023 using the PubMed and Web of Science databases (https://pubmed.ncbi.nlm.nih.gov/, https://clarivate.com/webofsciencegroup/solutions/web-of-science/). A search syntax strategy was devised using the following keywords: (“Brain diseases” OR “central nervous system diseases”) AND (“VNS” OR “Vagus nerve stimulator” OR “Vagal nerve stimulator” OR “Vagus nerve stimulation”). Articles were included if they were manuscripts published in English and described brain diseases in patients or animal research of brain disease models implanted with a VNS device for approved indications, and other articles were excluded.

## VNS induces anti-inflammatory effects in neurological disorders

**Fig. 1:**
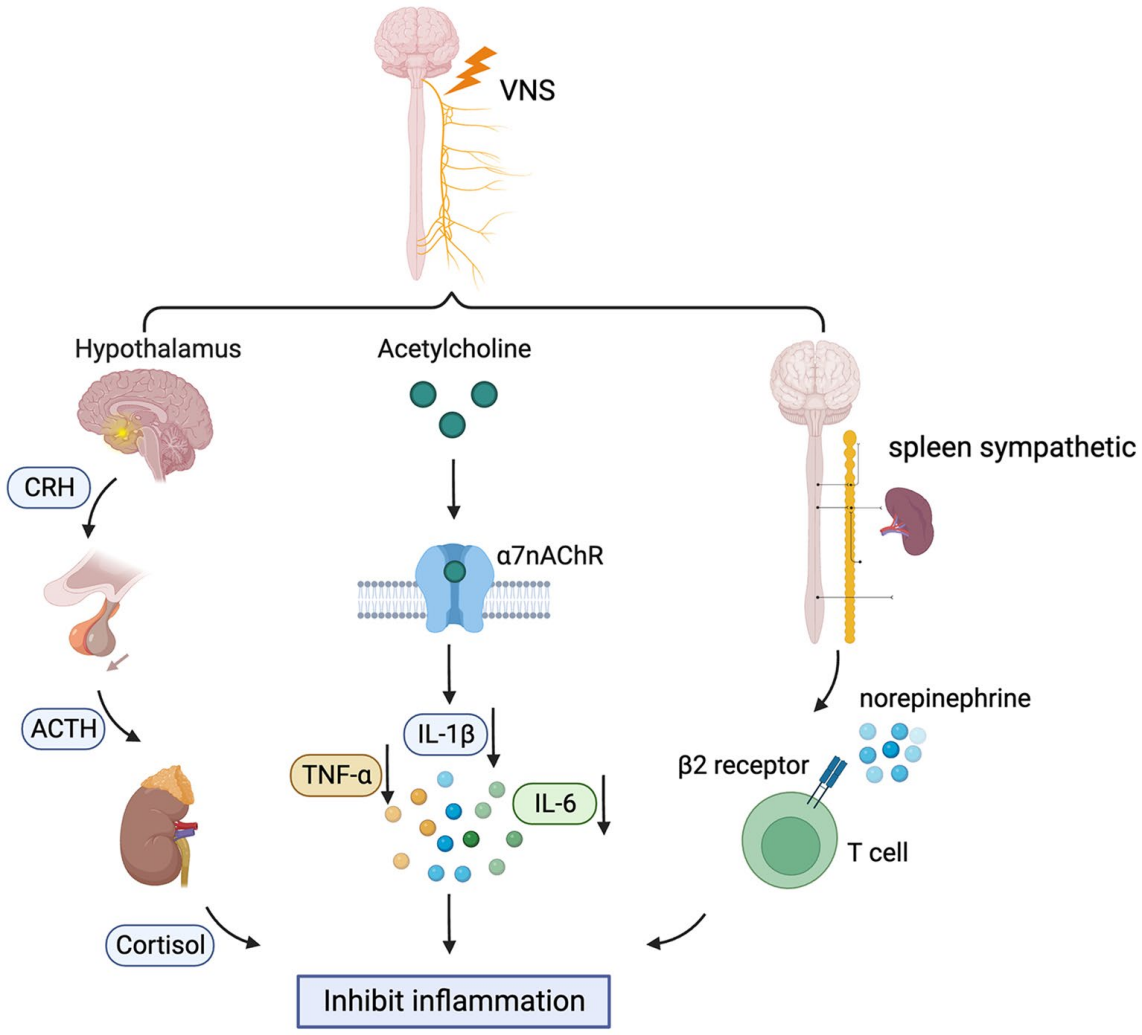
VNS inhibits inflammation mainly through three different mechanisms. VNS could increase cortisol levels along the HPA axis. The second way is by binding of acetylcholine to α7nAChR. Moreover, VNS acts on lymphocytes through splenic sympathetic nerves and inhibits inflammatory reactions. *VNS* vagus nerve stimulation, *α7nAChR* α7 nicotinic acetylcholine receptor, *IL-1β* interleukin 1β, *IL-6* interleukin 6, *TNF-α* tumour necrosis factor-α

The occurrence and development of CNS diseases are usually accompanied by inflammation [15]. Excessive inflammatory responses can promote the death of neurons and glial cells, leading to an aggravation of the condition [16]. The vagus nerve is known as a homeostatic component in the anti-inflammatory loop along the gut–brain axis. For example, in research focused on inflammatory bowel disease, VNS has been shown to regulate the intestinal inflammatory response. Chronic VNS (1 mA, 5 Hz, pulse width of 500 μs) was used to treat Crohn's disease model rats for 5 days in a previous study, which showed that VNS could reduce the extent of body weight loss and levels of inflammatory markers such as tumour necrosis factor-α (TNF-α) and interleukin-1β (IL-1β) [17–19]. Additionally, researchers are also applying VNS in the treatment of stroke, brain injury, depression, etc. [20]. As shown in Fig. 1, the results from these studies support the idea that VNS regulates inflammation through different mechanisms, as described below.

The hypothalamic–pituitary–adrenal (HPA) axis is one of the most studied pathways of VNS. Stimulation passes into the nucleus of the solitary tract through vagal afferent fibres, resulting in the release of corticotropin-releasing factor from the paraventricular nucleus of the hypothalamus, which initiates the HPA axis cascade reaction. The anterior pituitary gland produces and secretes adrenocorticotropic hormone, which induces the synthesis of glucocorticoids in the adrenal cortex [21]. A study of patients with depression demonstrated that VNS can increase glucocorticoid synthesis. There is glucocorticoid resistance in patients with depression, which leads to increased inflammation. Therefore, increasing glucocorticoids in patients with depression might inhibit the inflammatory response. An increased adrenocorticotropic hormone (ACTH) response to corticotropin-releasing hormone (CRH) challenge was found in patients with chronic depression. After 3 months of VNS treatment, serum cortisol levels were not significantly different from those of VNS-treated patients, but a reducing trend was observed [22]. Likewise, VNS treatment for 12 weeks could also reduce the peak value of serum ACTH induced by CRH in patients with depression [23]. An increase in serum cortisol is associated with mortality in patients with stroke, brain injury and other diseases. However, Herdt et al. found that serum cortisol was increased in rats undergoing acute VNS treatment [24]. Through the above clinical studies and animal experiments, we found that activation of the HPA axis is one of the potential mechanisms for VNS treatment. VNS can stimulate the nucleus tractus solitarius and hypothalamus through the vagus nerve and induce ACTH release and the synthesis of glucocorticoids. The increase in glucocorticoid levels may inhibit related inflammatory reactions. However, a downwards trend in serum cortisol was observed with long-term VNS treatment, which suggests that further research is needed on the HPA axis mechanism of VNS treatment, especially in terms of onset and duration. This is crucial for the treatment of different diseases in the future.

Another pathway is the cholinergic anti-inflammatory pathway, which is dependent on alpha 7 nicotinic acetylcholine receptor (α7nAChR). α7nAChR is a ligand-gated ion channel that has four transmembrane (TM) domains and a regulatory intracellular domain between TM3 and TM4 (counted from the N-terminus). The activation of α7nAChR by acetylcholine (ACh), nicotine or a specific agonist could recruit the tyrosine kinase Janus kinase 2 (JAK2) and then promote the phosphorylation of signal transducer and activator of transcription 3 (STAT3), through which the normal inhibitor of kappa B kinase (IKK) and nuclear factor kappa-B (NF-κB) inflammatory pathway could be inhibited, while the secretion of anti-inflammatory cytokines could be accelerated. It's worth noting that research primarily focuses on this route in the context of macrophages and monocytes [25]. α7nAChR is a key mediator in the pathologic changes of traumatic brain injury (TBI). Specifically, in a controlled cortical impact model of brain injury, a significant decrease in α7nAChR was found rapidly after trauma exposure (approximately 1 h), and the reduction could last for approximately 21 days, while a similar change was not found for any other type of nicotinic AChR or muscarinic AChR [26, 27]. Moreover, the reduction in TBI-induced reduction in α7nAChR also occurred in the hippocampus, which is distant from the injured cortex, indicating the existence of interbrain-area cholinergic connections in the injured brain [28]. In the fluid percussion injury (FPI) rat model, VNS continued for 14 days resulted in improved performance in the beam walk, forelimb reaching and flexion, and Morris water maze. Pathologic analysis indicated milder tissue loss (partly due to inflammation) and neurodegeneration [29]. Zhou et al. found reduced TNF-α levels in both the serum and brain tissue in animals with brain injury treated with VNS, suggesting that the activation of α7nAChR in macrophages might be important for recovery from TBI [30].

The middle cerebral artery occlusion (MCAO) model uses a monofilament nylon suture to block the middle cerebral artery and cause regional ischaemic for the purpose of simulating ischaemic stroke in humans. Rats with permanent MCAO (pMCAO), if treated with 1 h of VNS, had a reduced infarct area and increased phosphorylation of STAT3 and JAK2 in the ischaemic penumbra, while these beneficial effects were abolished in the α-bungarotoxin (α7nAChR antagonist)-treated VNS-pMCAO group [31]. In another study, 24 h after vascular occlusion in I/R mice, VNS reduced the release of proinflammatory cytokines, such as TNF-α, IL-1β, and IL-6, by activating α7nAChR in microglia and reduced the infarction volume [32]. This may be because VNS could upregulate the expression of peroxisome proliferator-activated receptor γ (PPARγ) by activating α7nAChR, which was confirmed to be able to exert anti-inflammatory effects on the CNS [33]. In addition, the study by Huffman et al. showed that after percutaneous VNS (pVNS) for mice with lipopolysaccharide (LPS)-induced endotoxaemia, the number of ChAT + /c-Fos + cells in the dorsal motor nucleus of the vagus nerve (DMX) was increased, and the level of TNF-α was downregulated, which indicated that pVNS restrained the expansion of inflammation and protected neural function in mice. Furthermore, they found that pVNS could modulate microglial activity in the hippocampus and reverse memory deficits induced by LPS [34]. In spinal cord injury, it was also found that VNS promoted the recovery of neural function through the α7nAChR anti-inflammatory pathway. Chen et al. found that in rats with spinal cord compressive injury, VNS treatment led to faster recovery of neural function after VNS, and it was notable that α7nAchR blockers could attenuate these effects. Further exploration in this study found that VNS could upregulate the shift of M1-polarized CD86 + microglia to M2-polarized CD206 + microglia via α7nAchR activation [35].

The third pathway is the spleen sympathetic and anti-inflammatory pathway. The major point of this theory is different from the other theories since it addresses the importance of stimulation of the efferent fibres of the vagus nerve. VNS induces ACh release from the celiac mesenteric ganglion, which promotes the release of noradrenaline from the spleen by binding with postsynaptic α7nAChRs of the splenic nerve [36]. Norepinephrine binds to the β2 receptor of splenic T lymphocytes and further releases ACh, which could inhibit the release of TNF-α by interacting with the α7nAChR of macrophages. There are currently few studies focusing on this pathway in CNS diseases, and more research is needed to clarify this specific upside-down modulation [37].

The α7nAChR found in macrophages and microglia play a critical role in facilitating inflammatory suppression during VNS treatment. Upon activation, these receptors amplify the levels of phosphorylated STAT3 and JAK2, which in turn inhibit the NF-κB pathway, a prominent inflammatory route. This inhibition consequently reduces the secretion of the main proinflammatory agents—TNF-α, IL-1β, and IL-6—by proinflammatory cells [25]. This process is pivotal in the capacity of VNS treatment to mitigate the CNS inflammatory response, preventing exacerbated inflammation following brain tissue injury. Moreover, VNS treatment fosters an increase in levels of PPARγ, known for its anti-inflammatory effects [38, 39], and catalyses the transition from M1-polarized to M2-polarized states in macrophages and microglia, further dampening the CNS's inflammatory response [40]. Nevertheless, current research predominantly concentrates on the central nervous immune system, often neglecting the peripheral distribution of the vagus nerve. Consequently, there is a limited depth of research and discussion on alterations in peripheral immune functions. While some studies have explored the modulation of splenic immune cells through the splenic nerve during VNS treatment, a comprehensive analysis of the relationship between peripheral and central immunity is lacking. This gap in knowledge signifies a potential new avenue for future research into the mechanisms of VNS.

## VNS has neural protective effects

**Fig. 2:**
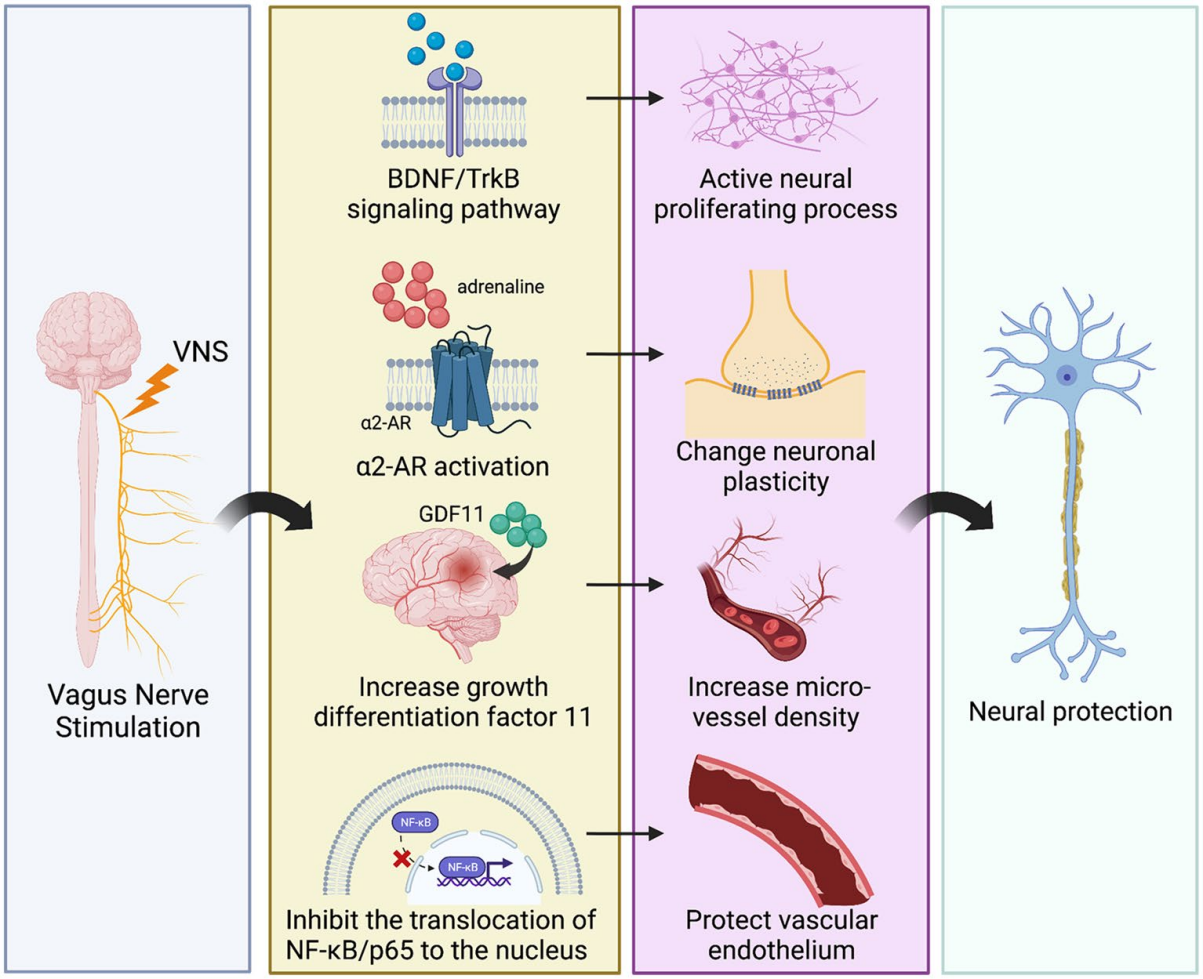
VNS can play a neuroprotective role through multiple strategies. VNS can activate the neural proliferation process via upregulation of the BDNF/TK pathway; it can activate α2-AR and change neural plasticity. VNS increases the level of GDF11, thus leading to an increase in microvessel density. In addition, VNS can at least partially inhibit the activation of the NF-κB pathway to protect vascular endothelial cells. *VNS* vagus nerve stimulation, *BDNF* brain-derived neurotrophic factor, *TrkB* tropomyosin-related kinase B, *α2-AR* α2 adrenergic receptor, *GDF11* growth differentiation factor 11; NF-κB, nuclear factor kappa-B

Previous studies have suggested that the vagus nerve plays an important role in neural protection, and multiple neuroprotective pathways of VNS were found, such as the release of neurotrophin, change in neuronal plasticity, and improvement in cerebrovascular regeneration (Fig. 2).

VNS can increase the content of brain-derived neurotrophic factor (BDNF), which is one of the most studied neurotrophins, to protect neurons via synaptic plasticity, inhibition of apoptosis, and enhancement of neural regeneration. The main receptor that mediates its biological activity is tropomyosin-related kinase B (TrkB). With the deepening of research, the role of the BDNF/TrkB signalling pathway has also been revealed in a variety of brain diseases, such as epilepsy and TBI [41]. Shah et al. found that after using K252a, an inhibitor of TrkB, the anxiolytic and antidepressant-like effects of VNS were inhibited, suggesting that one of the potential mechanisms of VNS involves the BDNF/TrkB signalling pathway. In addition, intraventricular administration of TrkB-Fc, a molecular scavenger for ligands of TrkB, blocked the antidepressant-like effects of VNS. This study indicated that the neuroprotective effect of increasing BDNF through VNS is ligand-dependent [42]. O’Leary et al. observed the brain tissue of mice one month after subdiaphragmatic vagotomy and found that BDNF mRNA in the CA1 and CA3 areas of the hippocampus decreased, which indicated that the intact vagus nerve maintained a normal level of BDNF in the hippocampus [43]. However, Lang et al. did not find changes in BDNF serum concentrations in patients with unipolar major depression, possibly because antidepressants had normalized BDNF release in serum prior to VNS treatment [44].

In MCAO rats, animals treated with VNS paired with rehabilitation training after brain vessel occlusion showed better forelimb performance than the rats treated only with rehabilitation training. This phenomenon was mainly due to the enhancement of the plasticity of the corticospinal motor network, thus further increasing the synaptic connectivity of the forelimb muscle [45]. In addition, in the research of Tseng et al. the rats were trained in a simplified version of the automatic lever pressing task. MCAO was conducted after the training. After 3–7 days of recovery after surgery, additional recovery training was carried out. Finally, internal simulation (ICMS) mapping of these rats was performed to obtain complete core motor maps. The combination of VNS and lever training significantly improved proximal forelimb (PFL) performance in the cortical motor maps. Moreover, injecting G-protein coupled α2 adrenergic receptor (α2-AR) antagonists into the primary motor cortex of MCAO rats prevented VNS-driven motor reorganization, which indicates that VNS enhanced neural plasticity depending on the activation of α2-AR [46]. On the other hand, VNS could also change neural plasticity in the nonmotor cortex. The brain’s default mode network defines a brain network involved in self-referential processing, affective cognition, and emotion regulation. In a study focusing on patients with depression, changes in the brain’s default mode network were found by using two 6-min resting-state functional magnetic resonance imaging (fMRI) scans after a month of noninvasive VNS treatment [47]. One possible explanation is that VNS might improve learning and memory abilities by enhancing the plasticity of the hippocampus [48]. Similar results were found in a study mentioning increased activities in the tractus solitarius and the locus coeruleus-central adrenergic system after VNS; these alterations might be correlated with the increase in long-term potentiation in the hippocampus [49]. Vargas-caballero et al. proposed that after the vagus nerve signal was transmitted into the locus basalis, it could secrete more catecholamines to act on neurons, astrocytes and microglia, which might enhance the plasticity and excitability of hippocampal, amygdalar and thalamic neurons [50].

In addition, VNS affected cerebral vascular regeneration. Male Sprague‒Dawley rats with focal cerebral ischaemia/reperfusion (I/R) injury had less severe neuronal damage in the penumbral area, which was correlated with increased microvessel density. The increase in endothelial cells with proliferating cell nuclear antigen PCNA/CD31 (two biomarkers of vascular proliferation) double staining can clarify this view [51]. In a similar study using I/R rats, the level of growth differentiation factor 11 (GDF11), a proliferation factor of brain capillary endothelial cells, was significantly increased after VNS in the ischaemic region, as well as in the plasma [52]. Additionally, the protective effects of VNS on vascular endothelial cells were found to be correlated with reduced activation of the NF-κB pathway [53]. Ovariectomized (OVX) rats were treated with chronic VNS, and inhibition of the translocation of NF-κB/p65 to the cell nucleus was observed in vascular endothelial cells by immunohistochemistry. Moreover, the results of light microscopy and electron microscopy showed that chronic VNS could prevent endothelial swelling, desquamation and even necrosis. This further demonstrated that chronic VNS could protect the vascular endothelium by inhibiting the inflammatory reaction mediated through the NF-κB/p65 pathway.

## VNS and protection of the blood‒brain barrier

Most CNS diseases, including brain tumours, brain injury, ischaemic stroke, and neurodegenerative diseases, are characterized by functional and structural changes in the blood‒brain barrier (BBB). Pathogen recognition receptors (PRRs), expressed on glial cells in the brain, bind to damage-associated molecular patterns (DAMPs), which allow glial cell activation and induce the release of inflammatory mediators and cytotoxic factors. The released inflammatory mediators, including cytokines, chemokines, reactive oxygen species (ROS) and lipid mediators, could further influence BBB integrity [54]. VNS could prevent the progression of cytokine leakage by protecting BBB integrity. First, previous studies showed that epilepsy was commonly accompanied by changes in the structure and function of the BBB [55, 56]. This may be one of the causes of cortical dysplasia-related refractory epilepsy [57]. In a study of kindled rats with cortical dysplasia, Kaya et al. found that after VNS treatment, the animals did not show either clinical or electrophysiological signs of seizures. They found the absence of horseradish peroxidase (HRP) reaction products in brain capillary endothelial cells of both the cortex and hippocampus [58]. In addition, Lopez et al. focused on the BBB integrity of TBI model rats. Vascular permeability was evaluated by xenogeny imaging (injection of FITC-dextran) and analysis of perivascular aquaporin 4 (AQP-4, an important protein related to BBB-mediated brain oedema) levels. They found that VNS significantly reduced FITC-dextran leakage and ameliorated brain oedema after TBI, which was correlated with reduced expression of AQP-4 [59].

One of the mechanisms by which VNS reduces BBB permeability is via α7nAchR regulation. VNS could inhibit the release of proinflammatory factors such as IL-1β, TNF-α and HMGB1 by upregulating α7nAChR in splenic macrophages, thereby protecting the BBB from destruction at the beginning stage [60]. In an ischaemic stroke model, rats with noninvasive VNS during the procedure of vessel occlusion had smaller infarctions and reduced BBB permeability in the lesion area, according to the dynamic contrast-enhanced MRI results. The immunomodulation of the microenvironment of the brain might be another explanation for VNS-induced protection of the BBB because reduced expression of metalloproteinases-2/9 (MMP-2/9) in astrocytes was found in the ischaemic area [61].

Alterations in the permeability of the blood‒brain barrier (BBB) are predominantly attributed to endothelial cell damage, heightened inflammatory responses, and a deficiency in neurotrophic factors. As previously noted, VNS treatment has the potential to augment the concentrations of neurotrophic factors within the brain [43, 44], a mechanism that potentially safeguards the BBB. However, this premise warrants further investigation. Concurrently, VNS serves to maintain the integrity of the BBB by curbing inflammatory responses, an action that complements the aforementioned inflammatory pathway. This treatment further dampens the activity of proinflammatory cells through the activation of α7nAChR, thereby diminishing the release of proinflammatory agents and playing a vital role in preserving BBB integrity [60]. Although numerous studies underscore the protective attributes of VNS on the BBB, the intricate mechanisms governing the interactions between immune components and vascular endothelial cells post-VNS remain somewhat elusive, necessitating further scholarly exploration.

## Multisystemic network of VNS in brain diseases

### VNS and microbiota–gut–brain axis

The microbiota–gut–brain axis describes the bidirectional signalling system between the CNS and gastrointestinal tract, with an emphasis on the importance of gut flora, which is composed of more than 10^14^ microorganisms, and over 100 times the amount of genomic content compared to the human genome [62]. The vagus nerve has both afferent and efferent fibres. Isolated stimulation of the vagus nerve could not only change the level of IL-1β in the hypothalamus and hippocampus [63], but also increase the activation of enteric glial cells, which is associated with improved intestinal barrier function [64]. Therefore, it should be noted that VNS is usually conducted on the cervical section as a whole, which could activate the vagus nerve both upstream and downstream; thus, the gut flora might be both the activator of the vagus nerve and the target of the vagus efferent output. In an animal study, the severity of LPS-induced encephalopathy could be attenuated by faecal transplantation from healthy donors, with milder brain inflammation and better behavioural performance, while these benefits did not occur if the vagus nerve lost its integrity [65]. Another example showed that the integrity of the vagus nerve was essential in the maintenance of immune balance after sleep deprivation-induced systemic sepsis because the immune improvement by faecal transplant from normal mice was abolished after subdiaphragmatic vagotomy [66]. On the other hand, Haney et al. conducted a one-hour intraoperative stimulation of the cervical vagus nerve in a mouse model of amyotrophic lateral sclerosis (ALS) and found no difference in gut bacterial composition between VNS and sham groups, indicating that the effects of a one-time VNS on gut flora might not last long and cannot alter gut flora; thus, chronic application of VNS might be recommended to change gut flora [67]. A recent study demonstrated that berberine, a chemical extracted from European barbery, once administered to mice, was able to induce more secretion of hydrogen sulfate (H_2_S) from gut microbiota. Extra H_2_S could activate the vagus nerve and ameliorate the behavioural deficiency in tMCAO rats, with reduced M1 and increased M2 polarization of microglia in the ischaemic cortex [68]. To date, only a few studies have addressed the vagus–microbiota–brain pathway, and the mechanism of VNS in brain disorders via modulation of gut flora seems underestimated and needs more attention.

### VNS and stress

Stress is one of the risk factors for AD, and several studies have found elevated cortisol levels in biological fluids such as plasma, saliva, and cerebrospinal fluid in patients with AD [69]. As previously mentioned, chronic VNS could effectively reduce serum cortisol levels, which therefore improved the prognosis of patients with AD by restricting stress severity. Cortisol could be reduced by VNS, thus postponing the development of AD, but research on the regulation of the HPA axis in patients with AD is still lacking [22]. Cortisol, in this specific case, is considered a double-edge sword. It not only inhibits the inflammatory response and improves the prognosis of diseases, but also acts as a factor in tissue damage. Therefore, how to regulate cortisol and its mechanism by VNS to limit the negative impacts of stress on individuals with brain disease still needs more study.

### VNS and obesity

VNS has the potential to influence the progression of obesity, a well-established risk factor for impaired cognitive function and the onset of conditions such as Alzheimer’s disease and ischaemic stroke. Gil et al. demonstrated that chronic VNS (10 ms, 200 mv, 1 Hz, and 10 Hz, respectively, for 12 h per day) reduced food intake, body weight gain, and epididymal fat pad weight in adult male rats fed a high-fat diet. In this study, the serum corticosterone concentration was significantly increased after VNS, and the number of c-Fos-positive cells in the NTS was increased as well. The activation of the HPA axis might be involved in these effects, as previously discussed [70]. In another study in rats, Banni et al. observed 50% and 80% increases in nonesterified fatty acid levels in plasma and mesenteric adipose tissue, respectively, after 4 weeks of VNS treatment, but VNS did not affect adipose levels in the liver. In the same study, VNS reduced the level of endocannabinoids while increasing levels of an endogenous ligand of the transcription factor PPARα in mesenteric adipose tissue but not in the hypothalamus, which suggested that VNS-induced reduction in body fat in rats might be the result of a combination of central and peripheral mediators [71]. In studies focused on humans, VNS was also found to increase the basal metabolic rate and energy expenditure, which might be related to brown adipose tissue (BTA) activation [71]. Therefore, chronic VNS, by limiting additional immune reactions, could increase energy consumption and has the potential to be a noninvasive treatment for weight loss in the future.

### VNS and the cardiovascular system

The VN is the longest cranial nerve in the body, with tight connections to different organs, including the cardiovascular system and respiratory system. VNS can not only directly act on the CNS to improve the outcome of brain diseases but also help to limit the progression of risk factors to limit the progression of diseases. Atrial fibrillation (AF) has been identified as one of the important risk factors for ischaemic stroke [72]. Yu et al. performed low-level vagal nerve stimulation (LL-VNS) in dogs with AF and found that VNS reversed the process of atrial electrical remodelling and reduced the risk of AF [73, 74]. In another study, AF animals in the LL-VNS group exhibited higher levels of STAT3 and lower levels of NF-κB in atrial tissue than the control group and the group administered methyllycaconitine (MLA) (an α7nAChR inhibitor). In addition, the levels of TNF-α and IL-6 in the LL-VNS group were lower, while the plasma level of ACh was higher in the LL-VNS group. These results showed that LL-VNS could inhibit atrial electrical remodelling by activating the α7nAChR-mediated cholinergic anti-inflammatory pathway because activated α7nAChR could inhibit the phosphorylation of NF-κB inhibitory protein, thus restricting the inflammatory response. At the same time, activated α7nAChR can lead to JAK2 phosphorylation and activate STAT3 in the downstream pathway, which plays an anti-inflammatory role [75].

**Fig. 3:**
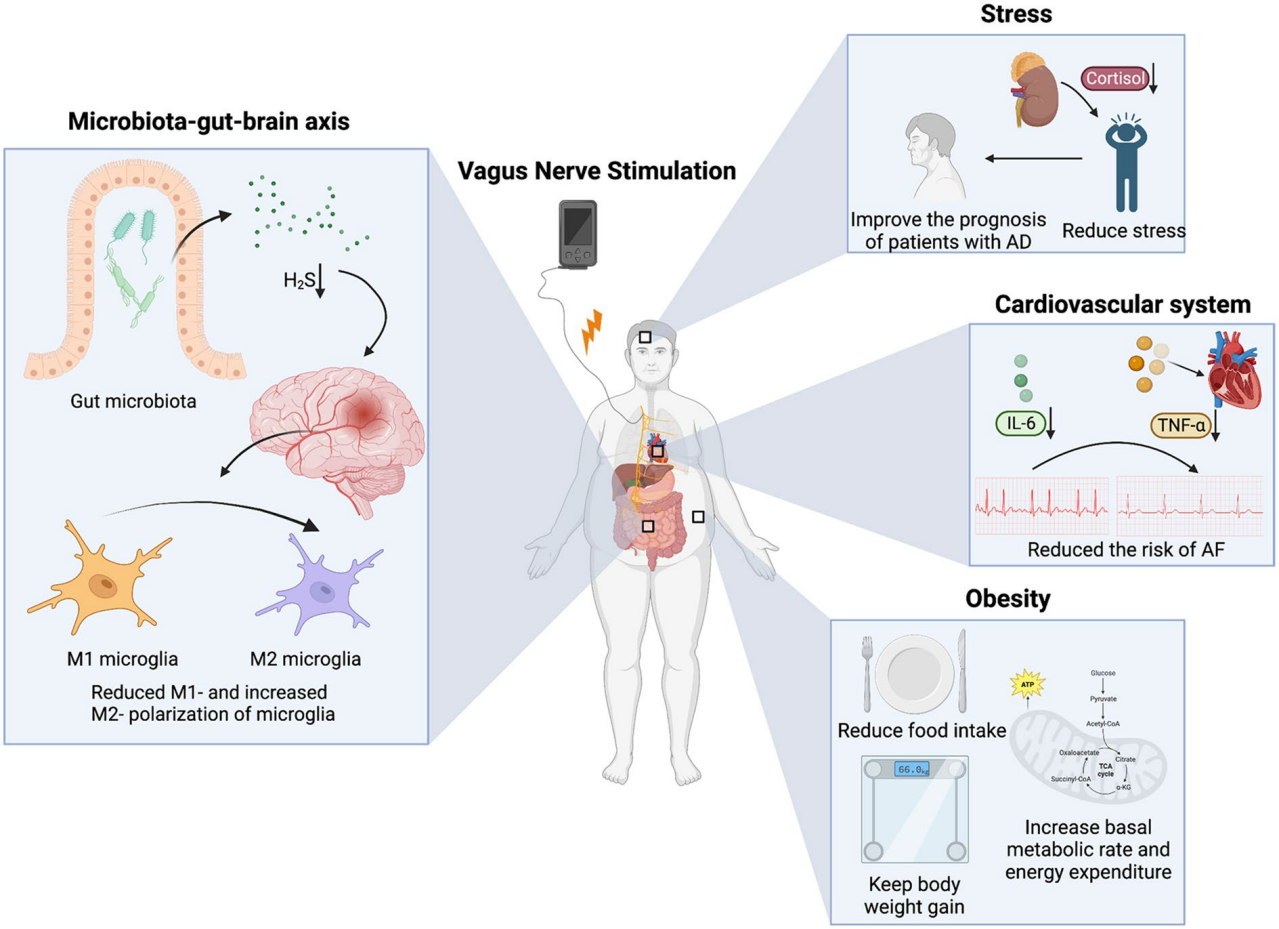
VNS improves outcomes of nervous system diseases through multisystemic modulation. One is via the microbiota–gut–brain axis because VNS can reduce the release of H_2_S by gut microbiota into circulation, further reduce the lesion severity of the nervous system, and cause an M2 shift in microglia. Other possible mechanisms include amelioration of atrial fibrillation and obesity, risk factors for ischaemic stroke. Furthermore, stress is known to have negative impacts on gut flora and induce poor prognosis of CNS diseases. VNS is able to reduce responses to stress, thus helping to stop the inflammatory loop along the gut–brain axis. *AF* atrial fibrillation, *IL-6* interleukin 6, *TNF-α* tumour necrosis factor-α

Evidently, owing to the extensive reach of the VN, VNS possesses the potential to improve or even prevent brain diseases through the modulation of a multisystemic network (Fig. 3). VNS treatment stands as a potent therapeutic and preventive approach for brain diseases, impacting not only the closely associated cardiovascular system, a critical player in cerebrovascular ailments but also addressing other risk factors pertinent to brain diseases. For instance, it can influence the gut–brain axis by altering the composition of the intestinal microbiota, modulating serum cortisol levels, and reducing the frequency of atrial fibrillation occurrences [73, 74]. Consequently, research delving into the mechanisms related to VNS should not be confined to the CNS. Future studies should encompass the distinctive anatomical structure of the VN, paying heightened attention to its interactions with both the CNS and peripheral systems, thereby offering a more comprehensive elucidation of the role VNS plays in regulating brain diseases. This review is subject to certain limitations. First, the majority of studies cited herein to delineate the mechanisms associated with VNS are based on animal models, with a noticeable absence of validation through clinical specimens. This highlights the necessity for sustained focus on clinical research concerning VNS and a deeper investigation into its underlying mechanisms in future studies. Second, although conditions such as anxiety, depression, and notably ischaemic stroke are emerging indications for VNS treatment, this article does not fully explore the potential adverse outcomes associated with VNS therapy for these diseases. While we have addressed some of the potential negative implications of VNS implantation and treatment, future literature reviews should delve deeper into the adverse reactions encountered in various disease populations, offering a broader and more balanced understanding of VNS's role in diverse medical contexts. Last, the scope of this review is somewhat constrained by the limited presence of meta-analyses and systematic reviews in the gathered literature, potentially compromising the depth and quality of the insights.

## Conclusion

In summary, as a neuromodulatory therapy, VNS holds positive impacts on neurological protection and improvement of disease prognosis in stroke, PD, AD, depression and other CNS diseases. The anti-inflammatory effect is considered to be positioned at the core of the whole map. Locally, VNS reduced the release of proinflammatory factors while promoting the release of anti-inflammatory factors, limiting the shift of microglia/macrophages to the M1 phenotype, promoting synaptic plasticity, and protecting the integrity of the BBB. In addition, due to its bidirectional nature, VNS could also regulate the inflammation of the CNS through restriction of peripheral immune reactivity. In addition, mainly via homeostatic effects, VNS could exert multisystemic modulation, including alteration of gut microbiota, and reduce the onset and progression of various types of risk factors correlated with CNS diseases. While VNS is a well-established treatment method, it has been associated with some adverse events, including potential cardiac issues and various side effects in patients treated for epilepsy. It is essential to consider these potential effects and to thoroughly assess a patient's physical condition before beginning treatment. The VNS treatment process usually includes long-term implantation and regular adjustments to parameters. More animal work and clinical trials are needed to identify important immune component(s) and pathways that VNS helps to regulate, which, by pharmaceutical administration, could mimic the therapeutic effects of VNS.

## Data Availability

These data were derived from the following resources available in the public domain: https://clarivate.com/webofsciencegroup/solutions/web-of-science/; https://pubmed.ncbi.nlm.nih.gov/. Any data associated with this review article will be made available by reasonable request to the corresponding author.
